# Digital Twins-Based Impact Response Prediction of Prestressed Steel Structure

**DOI:** 10.3390/s22041647

**Published:** 2022-02-20

**Authors:** Zhansheng Liu, Chao Yuan, Zhe Sun, Cunfa Cao

**Affiliations:** 1 Faculty of Architecture, Civil and Transportation Engineering, Beijing University of Technology, Beijing 100124, China; yuann@emails.bjut.edu.cn (C.Y.); zhesun@bjut.edu.cn (Z.S.); caocunfa@emails.bjut.edu.cn (C.C.); 2 The Key Laboratory of Urban Security and Disaster Engineering of the Ministry of Education, Beijing University of Technology, Beijing 100124, China

**Keywords:** digital twins, machine learning, impact response, prediction analysis, prestressed steel structure

## Abstract

Civil infrastructure O&M requires intelligent monitoring techniques and control methods to ensure safety. Unfortunately, tedious modeling efforts and the rigorous computing requirements of large-scale civil infrastructure have hindered the development of structural research. This study proposes a method for impact response prediction of prestressed steel structures driven by digital twins (DTs) and machine learning (ML). The high-fidelity DTs of a prestressed steel structure were constructed from the perspective of both a physical entity and virtual entity. A prediction of the impact response of prestressed steel structure’s key parts was established based on ML, and a structure response prediction of the parts driven by data was realized. To validate the effectiveness of the proposed prediction method, the authors carried out a case study in an experiment of a prestressed steel structure. This study provides a reference for fusion applications with DTs and ML in impact response prediction and analysis of prestressed steel structures.

## 1. Introduction

The operation and maintenance (O&M) of large civil infrastructure accounts for most of its total life span [1]. In recent years, large-span prestressed structures have been widely used in all kinds of public buildings (e.g., stadiums, exhibition centers, and transportation hubs) due to their reasonable force and strong practicability. Cable-bearing tension is the key part of prestressed steel structures [2]. In the O&M of structures (OMS), structure failures may occur due to terrorist attacks, natural disasters, explosions, and other accidental loads, resulting in catastrophic losses. Intelligent structural health monitoring is an important research direction for augmenting the current practice of O&M management with real-time monitoring, dynamic interaction, and automation techniques [3]. Among emerging information technologies, building information modeling (BIM), 3D laser scanning technology, and artificial intelligence (AI) concepts have been attracting increasing attention. Nowadays, most studies mainly focus on the environment, energy, and space. Cui et al. [4] presented a method that couples linear structures with three-dimensional surfaces to automatically reconstruct 3D models of indoor environments using point cloud data from mobile laser scanning. Zhao et al. [5] used 3D laser scanning to efficiently create the building energy model of existing buildings and to identify and evaluate the feasibility of existing building retrofitting schemes. Kim et al. [6] used BIM, 3D laser scanning, and intelligent algorithms for automated spatial analysis to help solve the problems derived from arrangement and installation of complex structures. However, few studies have examined the use of emerging information technologies for assessing structural safety under various impact loads.

An impact load is a complex dynamic process that generates a large amount of data and information. Determining the correlation between various coupling factors and structural impact responses for evaluating structure safety states is extremely challenging [7]. Although typical OMS could hardly predict the time, location, and intensity of accidental loading, scholars could control the time course of loading applied to the structure and the interaction form of the structure. At present, most studies mainly focus on experiments and numerical simulation for examining structures under various impact loads. However, these studies require tedious theoretical modeling efforts, high computing power, and continuous validation. Eagar et al. [8] collected information and investigated the ruins of the building caused by 9.11, seeking evidence of the building collapse. Eagar et al. studied the impact response of the whole structure by the impact resistance test. Wang et al. [9] explored the dynamic response of reinforced cable network structures under various impact loads and investigated the impact resistance performance of flexible cable networks. Unfortunately, limitations still exist. Simulating the actual condition of the structure under various working conditions by experiments is so far the most effective method. However, such experiments are often affected by various factors, such as the test equipment, measurement technology, and test methods, and often will cost a lot of time and money. Compared with the experimental method, the cost of finite element analysis (FEA) is lower. Still, some FEA methods are not practical in some cases (such as the dynamic finite element model). Discrete results, complex derivation processes, and uncertainties of boundary conditions create challenges when using FEA methods for practical engineering applications.

**Figure 1:**
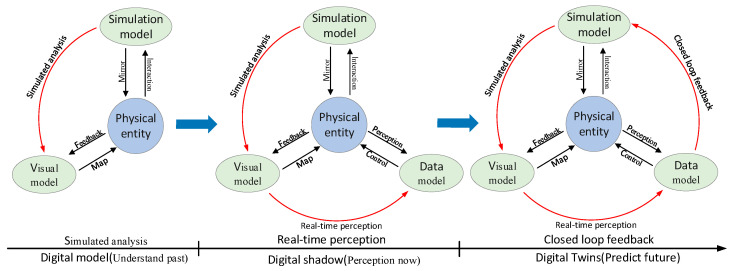
The evolution of DTs.

Industry 4.0, as an enabling force, will lead the development of the construction industry and revolutionize its practices and technologies [10]. DTs thus provide a new perspective for OMS by realizing the connection between digital/virtual spaces and actual physical spaces through real-time interactive data. Besides, DTs provide efficient, real-time, and intelligent services for the physical world [11,12]. Using DTs technology is an inevitable trend of development to optimize and improve OMS and mining the data generated in the OMS. There is currently confusion between the concept of building information modeling (BIM) and DTs, which may prevent the acceptance of DTs as a new concept [13]. DTs are significantly different from current digital models [14]. The construction and application of DTs are divided into three stages: digital model, digital shadow, and DTs (as shown in Figure 1). The digital model mainly includes physical entities, visual models, and simulation models. The visual model mainly realizes the visualization of physical objects in engineering design, construction, operation, and maintenance by using BIM technology. The simulation model analyzes and optimizes physical objects based on engineering experience and theory with the help of FEA. There is no efficient connection between the physical entity and the virtual model in this stage. The digital shadow is based on the digital model and data model. Digital shadow realizes the perception of physical entity states and parameters using sensors and other technologies. The initial connection between physical and virtual entities in digital shadows has been established, and real-time data can be synchronously updated to the virtual model. Based on digital shadow, the DTs make full use of intelligent algorithms such as machine learning to conduct in-depth mining of field data and simulation data. DTs finally realize the prediction of the future state of the physical entity and provide key decision information for construction engineering management.

As a new mode of digitally copying and simulating the behavior of physical objects, DTs can provide real-time stress, strain, and safety status of structures during operation and maintenance through data acquisition of sensor technology. DTs can evaluate the safety state of OMS and provide effective decision information by using intelligent algorithms. However, only limited studies have investigated DTs in predicting structural impact response. The main challenges involved in this include: (1) the fact that no mature method exists to construct the DTs model of prestressed steel structure; (2) that there is no detailed description of the predicted structural impact response process; and (3) that there is no reliable algorithm to predict structural impact response.

This study proposes an impact response prediction method for prestressed steel structures driven by DTs and ML. A high-fidelity DTs model of a prestressed steel structure is constructed from both the physical entity and virtual entity to realize real-time visualization of the structure’s state. This paper presents an improved neural network algorithm to examine the structural impact response under multiple complex factors. Finally, the authors carried out structural simulation and test experiments for validating the feasibility of the proposed method. Results indicate that the proposed method could guide the OMS and improve the data-driven mechanism of the DTs-based impact response prediction analysis. Additionally, this study provides an analytical basis for the state evaluation of prestressed steel structures.

The rest of this article is organized as follows. Section 2 provides a literature review concerning structural response prediction and DTs. Section 3 introduces the proposed DTs-based framework for structural impact response prediction. Section 4 describes the structural impact response prediction analysis method driven by DTs and ML. Section 5 uses a case study for validating the proposed method. Section 6 summarizes the major conclusions of this study.

## 2. Literature Review

### 2.1. Structural Response Prediction

Structural response simulation is an ideal method to evaluate structural performance under load [15]. As a complex dynamic process, the impact makes it difficult to determine the correlation between coupling factors, structural state, and impact response. Although there have been more and more tests and numerical reports on impact loads, there is still a lack of suitable prediction models. Traditional prediction models such as dynamic finite element models are not practical in some cases. Besides, the complexity of modeling and high computational cost hinders the efficiency of FEA. FEA cannot meet the requirements of timely data exchange in the OMS. ML methods such as the artificial neural network (ANN), Bayesian network modeling, and support vector machine (SVM) models have been widely used in the research and practice of structural engineering to improve the effectiveness of processing capability and prediction results [16]. Since the mid-1990s, ANN has been gradually applied to structural health monitoring, damage identification, and structural response prediction [17,18,19,20]. Lam et al. proposed a pattern recognition method for structural health monitoring based on ANN [21]. Quoc et al. used a neural network to predict the damage modes of the reinforced concrete slab under impact load using a small dataset [22]. In addition, ANN can be used for structural safety assessment [23,24]. These studies illustrate the potential of ANN in solving different civil engineering problems.

### 2.2. Digital Twins (DTs)

NASA’s Apollo program proposed the concept of DTs to predict the remaining life and health maintenance of spacecraft [25]. Based on Grives’ 3D model [26], Tao et al. proposed a five-dimensional DTs model and explored the main potential application fields of this model [11]. DTs are now widely used in architecture, engineering, construction, and operations (AECO). For example, Grigor Angjeliu [27] has developed a program to establish accurate DTs models for investigating the structural response of ancient buildings and the preventive maintenance measures. Liu [28] et al. proposed a DTs-based indoor safety management system framework, which utilizes the Internet of Things (IoT), BIM, and SVM to improve the intelligent level of building indoor safety management. Yu [29] et al. established a decision-making analysis framework for tunnel O&M based on DTs, providing reasonable and feasible countermeasures for guiding and optimizing O&M management. Jiang [30] et al. discussed the application of DTs to the fatigue life prediction of steel bridges. In addition, some people have combined FEA and BIM to build DTs models verifying the feasibility of building a high-fidelity model [31,32]. However, the implementation details of the DTs and their application in building O&M need further exploration.

## 3. Framework for DTs-Based Structural Impact Response Prediction

### 3.1. Five-Dimensional DTs Models

Referring to Tao Fei’s five-dimensional DTs model [11], this study proposed a structural O&M management model based on DTs. Data exchange between the physical and virtual world is realized. The data includes structural BIM information, FEA information, 3D point cloud information, and real-time structural O&M information. This paper realizes the precise mapping from the physical world to the virtual world with the help of DTs. This study explores multiple dimensions of OMS management and defines the information depicted by the DTs models of OMS. The models can be defined as *M*_DTs_, which is shown in Formula (1):

(1) $$M_{\mathrm{DTs}}=(PM,VM,CN,DD,AS)$$

**Figure 2:**
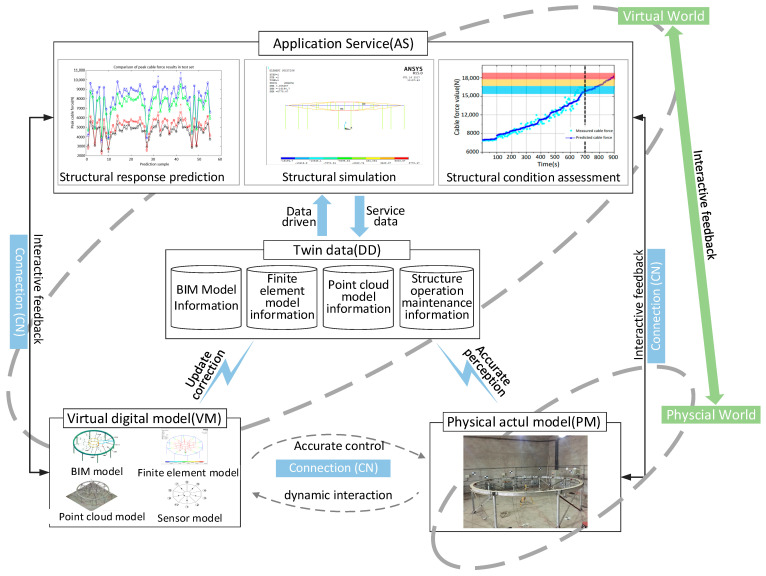
The DTs-OMS model of prestressed steel structure.

*PM* refers to the actual physical model, which is the foundation of DTs. *VM* exponential virtual model is the faithful mirror of *PM* in the digital space and evolves synchronously with *PM*. *CN* refers to the connection between the parts of *M*_DT_; *DD* refers to physical data, simulation data, and merging data. *AS* refers to various service functions in the structural O&M stage, such as predicting security risks, visualizing structural states, and providing simulation services. The model framework is shown in Figure 2.

### 3.2. Framework for Impact Response Prediction of Prestressed Steel Structures

**Figure 3:**
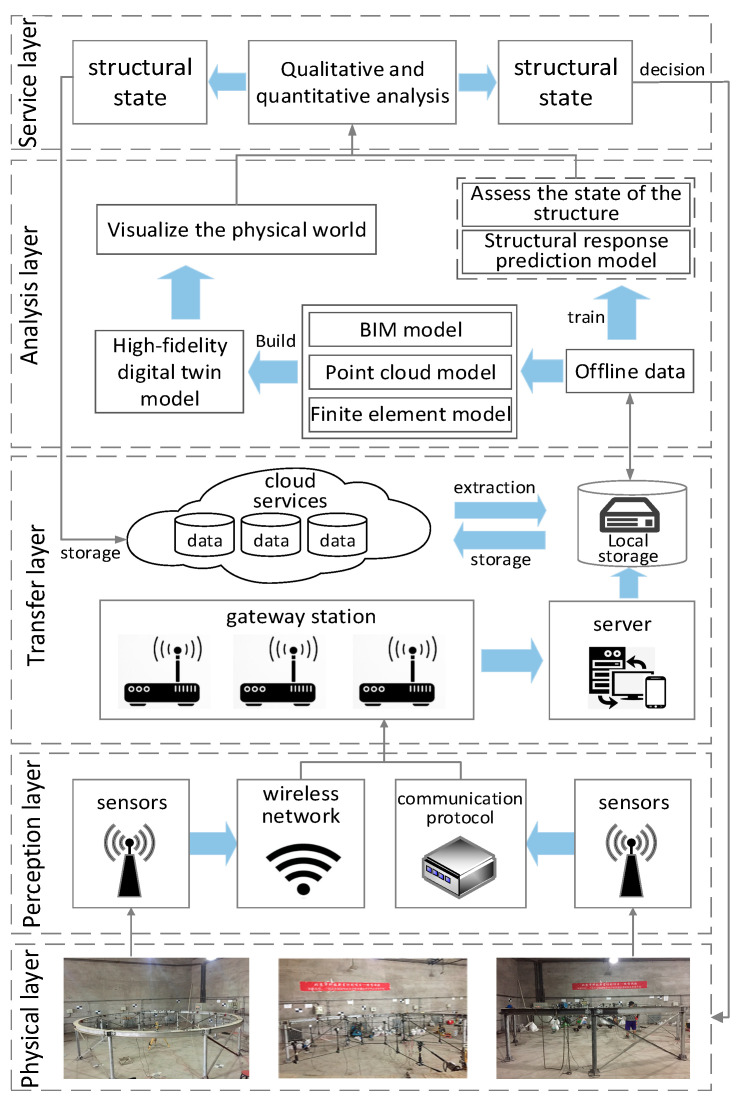
The overall framework for impact response prediction of structures.

The framework for impact response prediction of prestressed steel structure mainly includes the physical layer, perception layer, transmission layer, analysis layer, and service layer (as shown in Figure 3).

The physical layer contains the prestressed steel structure, which is the main object and basis of impact response prediction and provides the information of prestressed steel structure entity attribute, real-time mechanical state, environmental parameters, etc. for the perception layer.

The perception layer mainly collects twin data and provides the basis for the operation of the DTs model. Twin data covers the life cycle information of the structure as well as relevant specifications and experience in the impact field. The twin data are the direct embodiment of the property of structure entity, real-time mechanical state, and environment. Twin data have a strong spatial and temporal correlation, and they influence and interact with each other, presenting a cycle and iterative process.

The transmission layer mainly transmits the structural data collected from the perception layer to the analysis layer to realize data access, transmission, and storage. This layer is the link to realize the virtual-real interaction. After the perception layer collects twin data, the transmission layer is transmitted to the analysis layer through high-speed, high stability, and low delay data transmission protocols (e.g., HTTP, SNMP, FTP, etc.). In this way, the stability and robustness of data transmission are guaranteed to enable interactive feedback between the physical and the virtual world.

The analysis layer uses DTs to visualize the real-time state of prestressed steel structure in the physical layer. This study uses the twin data-driven machine learning model to mine massive heterogeneous information and realizes the structural impact response prediction analysis driven by the combination of DTs and ML.

The service layer is the “window” necessary to realize the prediction analysis of OMS. The BIM service platform visualizes the state prediction results and then implements predictive maintenance measures for the structure.

## 4. Structural Impact Response Prediction Analysis Method Driven by DTs and ML

### 4.1. Construction of DTs Based on Virtual and Real World

#### 4.1.1. Physical Entity Modeling

The physical entity of prestressed steel structure includes all kinds of multi-source heterogeneous total factor information in the physical world, mainly including structure entity, sensor entity, and surrounding environment. First, the study utilizes advanced sensing technologies to sense structure entity states in real-time, including the basic properties of temperature, humidity, load, stress, and strain in OMS. Second, the massive sensing data in the dynamic network environment is transmitted to the network end in real-time, and control and scheduling commands are accurately fed back as required. Sensing technologies realize the intelligent perception and interconnection, efficient transmission and integration, real-time interaction, and control of all elements in the physical scene. Finally, the recorded data are used for intelligent prediction and collaboration of mechanical models, geometric models, actual monitoring data, and safety information in OMS.

**Figure 4:**
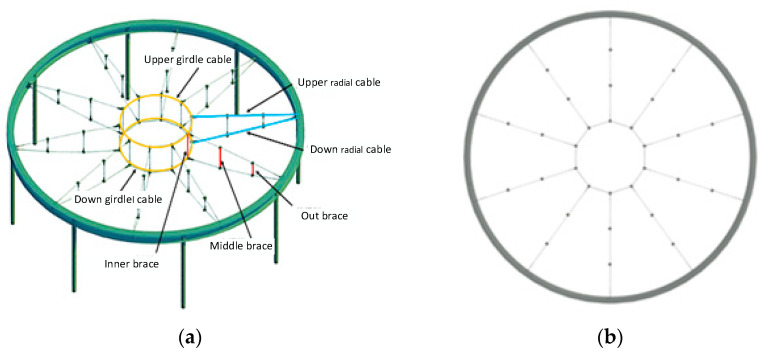
The model of the structure. (**a**) 3D model of the structure; (**b**) Plane graphs of the structure.

The model test uses a prestressed steel structure with a geometric scale ratio of 1:10 compared with the actual structure. The cross-sectional area ratio of cable and brace in the physic engineering structure is 1:100. The materials used in the test are the same as the actual structure. The structure needs mass compensation to meet the principle of the 1:1 stress ratio between the scaled model and the actual structure model. According to the calculated nine times compensated dead weight load, the joint force is applied to the nodes of the scale model. The experimental model is 6 m in diameter and consists of 10 trusses of radial cable net structure, two ring cables, and an outer ring I-beam. Figure 4 shows the 3D diagrams and plane graphs of the structural model.

**Figure 5:**
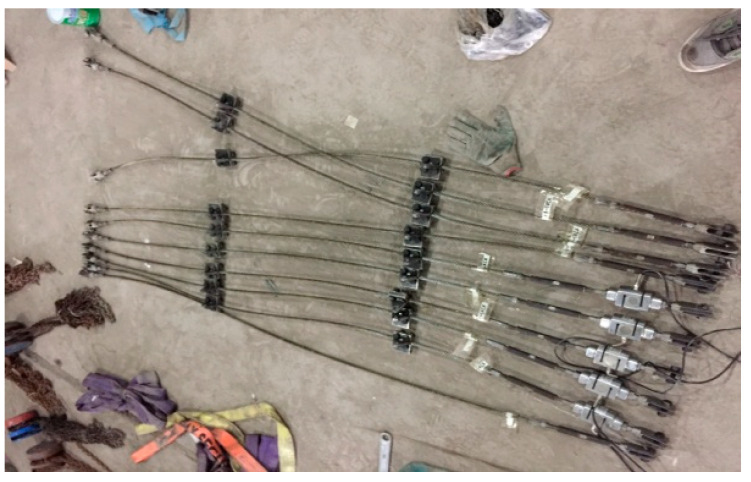
The diagram of the cable structure.

On the premise of the structural design drawings, the structural test model is calculated and analyzed, and the selected model materials and specifications are shown in Table 1 in combination with the actual market supply situation. The authors adopted various rod and node forms of the structural test model, considering the mechanical property of the structure. The cable level is 1570, and the cable structure diagram is shown in Figure 5.

#### 4.1.2. Virtual Entity Modeling

**Figure 6:**
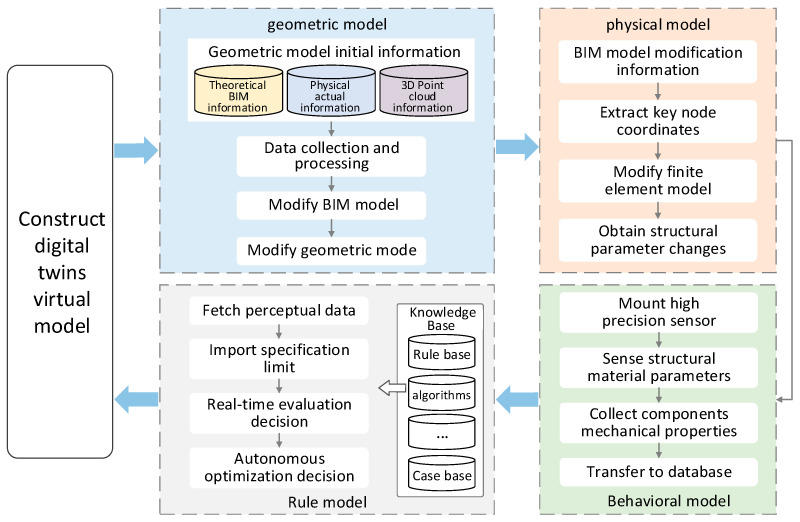
Construction of high precision DT_S_ model.

The study constructed a multi-dimensional and multi-scale virtual model from dimensions of geometry-physics-behavior-rules to realize precise mapping from the physical to the virtual world (see Figure 6).

The geometric dimension mainly applies BIM software for establishing the structural geometric model. During the model test, the test model is often different from the designed test model, so it is necessary to establish a structural geometric model with high-fidelity. First, the authors have established a theoretical BIM model and FEA model in the design stage. Second, the authors used 3D laser scanning technology to scan the test model in the experiment and collected 3D point cloud data that contains 3D coordinates of the actual structure. The authors then imported the 3D point cloud data into BIM software to obtain the coordinates of key nodes of the structure. Finally, the BIM model is modified according to the above node coordinates, and a high-precision geometric model is established. The geometric model of prestressed steel structure can be established at a low cost using 3D laser scanning technology. The establishment of the high-fidelity geometric model can provide a solid foundation for subsequent physical model analysis.

**Figure 7:**
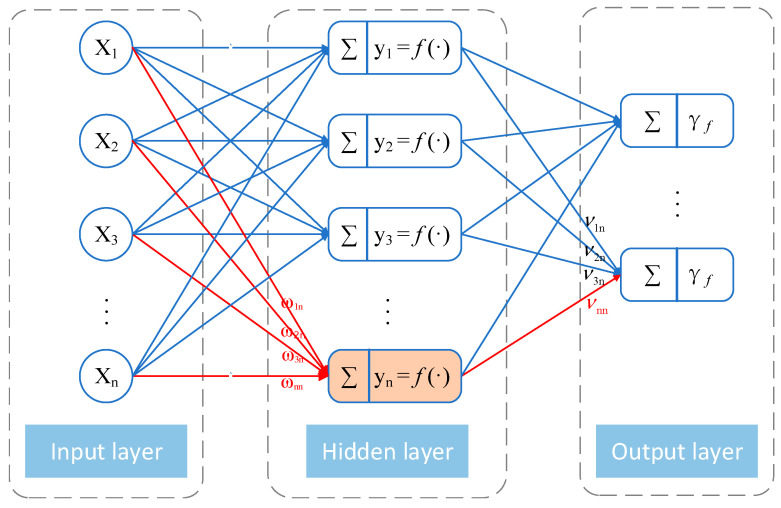
Topological structure of BP neural network model.

The physical dimension mainly describes the physical properties of the structure by using finite element simulation software, such as the material parameters and mechanical properties of structural components. Based on the modified BIM model in the geometric dimension, this test established a modified finite element model considering the local error in the O&M stage to ensure the accuracy of the simulation analysis. The analytical data can better reflect the static performance of the actual test model. The linear elastic structure analysis software ANSYS/LS-DYNA was used to establish a high-fidelity 3D finite element model of prestressed steel structure, as shown in Figure 7. Meanwhile, using ANSYS Parametric Design Language (APDL) establishes the structure model. The study uses the link160 element to simulate the girdle member, the link167 element to simulate the cable, and the solid 164 elements to simulate the mass block. Applying initial strain to the cable element simulates the prestress.

The behavioral dimension is mainly about real-time data perception of the whole physical structure through high-precision sensor devices. In this test, high-precision sensors and measuring equipment are installed on the key components of the cable network structure. For example, magnetic flux sensors for measuring boundary main cable forces are installed on upper and lower radial cables and upper and lower ring cables. The actual information changes of material parameters and mechanical properties in the OMS can be collected and transferred to the database for storage.

The rule dimension quantifies the physical and mechanical parameters of structural components, according to national standards, intelligent algorithms, and case experiences. This test determines the minimum breaking pull of cable according to the standard “General Technical Conditions for Steel wire Rope” GB/T20118-2017. According to “General Technical Conditions for Steel Wire Rope” GB/T20118-2017, the minimum breaking pull of upper radial cable and upper girdle cable is 33.4 kN, and the minimum breaking pull of lower radial cable and lower girdle cable is 48.2 kN and 69.4 kN. The conversion relation between allowable stress and minimum breaking stress of cable is shown in the Formula (2). The conversion factor is 0.88 and 0.85, and the safety factor is 4.

(2) $$F_{allowable}=F_{break}\times conversion\;factor/safety\;factor$$

After calculation, *F_allowable_* (upper radial cable, upper girdle cable) is 7348 N. *F_allowable_* (lower radial cable) is 10,242 N. *F_allowable_* (lower girdle cable) is 14,747 N.

In addition, the digital communication between the OPC and PLC module of MATLAB is used to obtain timely data feedback and ensure the dynamic interaction of virtual and real space. In this way, each round of data takes less than 1 s. The new data obtained each time will be stored in the.txt file of the specified folder, replacing the old data file. Therefore, ANSYS carries out real-time simulation analysis by calling.txt file.

### 4.2. Structural Impact Response Prediction Method Based on ML

#### 4.2.1. Levenberg-Marquard Algorithm

Levenberg-Marquard (LM) algorithm is a function that minimizes function values by finding parameter vectors. The basic formula of the LM algorithm is:

(3) $$x_{k+1}=x_{k}-\frac{\nabla F(X)|x=x_{x}}{\nabla^{2}F(X)|x=x_{x}}$$

where *x_k_* and *x_k_*_+1_ are gradients. Let the error function adopted by BP neural network be:

(4) $$e_{k}=\frac{1}{2}{\left(y_{lk}-{\bar{y}}_{lk}\right)}^{2}$$

In the above Formula (4), *e_k_* represents the error, *y_lk_* represents the expected output of the set, and *y_lk_* represents the actual output of the calculation. The performance index *F*(*x*) adopted by the network can be expressed as:

(5) $$F(x)=\sum_{i=1}^{N}e_{i}^{2}(k)=E^{2}(x)E(x)$$

Therefore, the gradient component of the number *j* is:

(6) $$\nabla F{\left(x\right)}_{j}=\frac{\partial F(x)}{\partial x_{j}}=2\sum_{i=1}^{N}e_{i}(x)\frac{\partial e_{i}(x)}{\partial x_{j}}$$

The gradient matrix form is:

(7) $$\nabla F(x)=2J^{T}(x)E(x)$$

*J^T^*(*x*) is the transpose Jacobian.

LM algorithm formula is as follows:

(8) $$x_{k+1}=x_{k}-{\left[J^{T}(x_{k})J(x_{k})+\mu_{k}I\right]}^{-1}J^{T}(x_{k})E(x_{k})$$

#### 4.2.2. Back Propagation Neural Network Algorithm

The back propagation (BP) neural network algorithm is a multi-layer feedforward network trained according to error backpropagation [33], including input layer, hidden layer, and output layer. At present, the research and application of the BP algorithm in various subjects develop rapidly. The BP algorithm is suitable for fitting all kinds of complex nonlinear relations. The BP algorithm’s main advantage is that it does not need to guide the mechanism model that the input and output variables satisfy, but to fit by itself through a large number of sample data. The topological structure of the BP algorithm model is shown in Figure 7. BP algorithm can build a mathematical model that describes the mapping between inputs and outputs [34]. In addition, Zhang et al. [35] proved that the three-layer BP model has sufficient accuracy for the approximation of nonlinear functions. BP algorithm includes two processes: feedforward and backpropagation. In the feedforward process, neurons produce output through “activation function” processing. If the actual output of feedforward does not match the expected, the error propagates back. The backpropagation of the error adopts the gradient descent method, so the minimum value of the function can be found faster by verifying the opposite direction. In backpropagation, the output error is transmitted back to the input layer through the hidden layer, and the error is apportioned to each unit. Then, the parameters corresponding to the minimum error are determined by adjusting the connection strength between the input node and the hidden node and the connection strength between the hidden node and the output node and adjusting the threshold. Finally, by changing the connection weights and thresholds of the network to adapt to the external environment, to meet the error requirements, output the best-predicted value. Therefore, the BP algorithm has the ability of fault tolerance, learning, and adaptation.

The output error of neural network is defined as follows:

(9) $$E=\frac{1}{2}{\left(D-\gamma \right)}^{2}=\frac{1}{2}{\sum_{i=1}^{i=m}\left(d_{i}-\gamma_{i}\right)}^{2}$$

Expand from the hidden layer to the input layer. To follow the principle for error reduction, the weight adjustment should be as follows:

(10) $$\Delta W_{ji}=-\alpha \frac{\partial E}{\partial W_{ij}},j=1,2,\cdot \cdot \cdot ,m;i=1,2,\cdot \cdot \cdot ,l$$

(11) $$\Delta V_{kj}=-\beta \frac{\partial E}{\partial V_{ij}},k=1,2,\cdot \cdot \cdot ,n;j=1,2,\cdot \cdot \cdot ,m$$

In this way, the calculation formula for adjusting the weight of each layer can be obtained. The written vector is in the form of:

(12) $$\Delta V=\eta {\left(\delta^{y}X^{T}\right)}^{T}$$

(13) $$\Delta W=\eta {\left(\delta^{o}X^{Y}\right)}^{T}$$

In the Formula (14), *X* = (*x*_1_*, x*_2_*, x*_3_*,…, x_n_*) is the input vector, *Y* = (*y*_1_*, y*_2_*, y*_3_*,…, y_n_*) is the output vector of the hidden layer, γ = (γ_1_, γ_2_, γ_3_,…, γ_n_) is the output vector, *D* = (*d*_1_*, d*_2_*, d*_3_*,…, d_n_*) is the expected output vector, *W* = [*W_ij_*]*_m×l_* and *V* = [*V_ij_*]*_m×l_* are the connection weight matrices from the hidden layer to the output layer and from the input layer to the hidden layer.

The learning and training process of the BP algorithm has four steps, which are as follows:

(1) The network is initialized, and the weight coefficients are randomly selected manually while the parameters of the BP algorithm are assigned;
(2) Input the learning and training data samples, calculate the estimated value of each layer of the network, and compare with the measured value. Calculate the output error of the algorithm;
(3) According to the rule of error backpropagation, the weights between hidden layers and between hidden layers and input layers are adjusted.
(4) Repeat steps 2 and 3 until the final prediction error meets the predetermined error range or the training number of the network reaches the predetermined number.

#### 4.2.3. LM-BP Neural Network Model

The traditional BP algorithm mainly has defects, such as being easy to fall into paralysis, a slow convergence speed, being easy to fall into the local optimal solutions, and not getting the global optimal solutions. The improved algorithm overcomes these two shortcomings [36]. LM algorithm is a variant of the Gauss-Newton algorithm for solving nonlinear least-squares problems. The LM algorithm combines the Gauss–Newton method with the gradient descent method. Therefore, LM algorithm has both local features of the Gauss–Newton method and global features of the gradient descent method. The LM algorithm can effectively prevent the BP algorithm from getting global optimal solutions due to entering local minimum. The LM algorithm has fast convergence speed and high efficiency, suitable for solving function approximation problems.

After the *t*_0_ output, the total error data are obtained:

(14) $$e(i):={\left[e(i),\cdot \cdot \cdot ,e(i-t_{0}-1)\right]}^{T}$$

(15) $$e(i):=J{\left(i\right)}^{T}{\tilde{\theta }}_{i}+\eta (i)$$

And then get

(16) $$J(i):=\left[\xi (i),\cdot \cdot \cdot ,\xi (i-t_{0}-1)\right]$$

(17) $$\eta (i):=\left[\eta (i),\cdot \cdot \cdot ,\eta (i-t_{0}-1)\right]$$

The above formulas are regarded as linear equations and obtained by Gauss–Newton method:

(18) $${\tilde{\theta }}_{i}={\left(J(i)J{\left(i\right)}^{T}\right)}^{-1}J(i)e(i)$$

However, there is no guarantee that any *t*_0_ and *x*(*i*) are full ranks. Therefore, according to the improved Gauss–Newton LM algorithm, *J*(*i*)*J*(*i*)*^T^* is replaced by *J*(*i*)*J*(*i*)*^T^* + *μ*(*i*), (*μ*(*i*) > 0), and the learning algorithm is replaced by:

(19) $${\tilde{\theta }}_{i}={\left(J(i)J{\left(i\right)}^{T}+\mu (i)I\right)}^{-1}J(i)e(i)$$

Finally, the *σ* parameter correction algorithm is added to obtain the final error learning algorithm:

(20) $${\tilde{\theta }}_{i+1}={\tilde{\theta }}_{i}-{\left(J(i)J{\left(i\right)}^{T}+\mu (i)I\right)}^{-1}J(i)e(i)-\sigma {\tilde{\theta }}_{i}$$

In this way, the weights of each layer can be continuously corrected and adjusted through error backpropagation until the output error w of the network is reduced to a preset acceptable level. The improved BP algorithm is applied to predict the impact response of prestressed steel structures in order to improve the safety performance of OMS.

#### 4.2.4. Structural Impact Response Prediction Flow Based on LM-BP Algorithm

The impact is a complex dynamic process that cannot be easily described by a reliable mathematical formula. The correlation between coupling factors and characteristic impact response is difficult to determine [7]. Therefore, it is hard to establish a reliable mathematical relationship between multiple coupling factors and impact response. Neural network algorithms can discover correlations between independent and dependent variables through computations based on large samples, thus achieving reliable classification and regression. This paper uses the improved BP algorithm to mine the structural data and obtain the trained algorithm model applied to predict the dynamic response of impact. The developed model needs input variables to simulate structure under the impact, such as the percentage of residual prestress from different members (%), impact height and impact mass, etc. The proposed model can give simulation results such as peak displacement (mm) and peak impact force (N) by inputting the corresponding input variables into the model. However, the simulation results are data points, not time course results.

The steps of impact response prediction based on the LM-BP algorithm are as follows:

(1) Data preprocessing. There is a pre-processing process to reduce the impact caused by randomness and diversity of dynamic responses, such as normalization. The input and output matrices of the training and test set are normalized, and each normalized value is normalized to the interval [0, 1]. The conversion equation is shown as (21).
  (21) $$x=\frac{\left(x-\min\right)}{\left(\max-\min\right)}$$
(2) Selection of training and test set. LM-BP algorithm is developed by MATLAB. According to the proportion of 70%, 15% and 15%, the test dataset was randomly divided into a training set, validation set, and test set.
(3) Model building and validation. The neural network is initialized by assigning random values to connection weights and thresholds. The network is trained until the model performance evaluation index meets the predetermined termination criteria or exceeds the maximum training times. After the training is complete, the optimal connection weights and thresholds are sent as outputs ready for use in the prediction section. When given new inputs, the prediction part can predict the outcome based on the basic knowledge gained from the training part.
(4) Evaluation of prediction models. To evaluate the prediction performance of the LM-BP model, several statistical criteria shown in Formulas (22)–(24) were selected as the evaluation index values of the prediction model performance, including the goodness of fit (*R*^2^), mean squared error (*MSE*), and mean absolute error (*MAE*).

The goodness of fit *R*^2^ is the standard for evaluating the good fit of the regression model. Its value varies between [0, 1]. The closer the function value is to 1, the better the model fitting effect is. The expression for *R*^2^ is

(22) $$R^{2}=\sum_{i=1}^{N}{\left(t_{i}-o_{i}\right)}^{2}/\sum_{i=1}^{N}{\left(o_{i}-o_{avg}\right)}^{2}$$

where *o_i_* and *t_i_* are, respectively, the predicted value and the actual value of the *i*th sample. The *o_avg_* is the average of the predicted output and *n* is the number of all samples.

The *MSE* is used to measure the error between the predicted value and the actual value. If the *MSE* approaches 0, the predicted value is close to the actual value. The expression for *MSE* is

(23) $$MSE=\frac{1}{N}\sum_{i=1}^{N}{\left(t_{i}-o_{i}\right)}^{2}$$

The *MAE* not only considers the error between the predicted value and the actual value but also involves the proportion of the error. The expression for *MAE* is

(24) $$MAE=\frac{1}{N}\sum_{i=1}^{N}\frac{\left|t_{i}-o_{i}\right|}{t_{i}}$$

## 5. Case Study

### 5.1. Experiment Description

**Figure 8:**
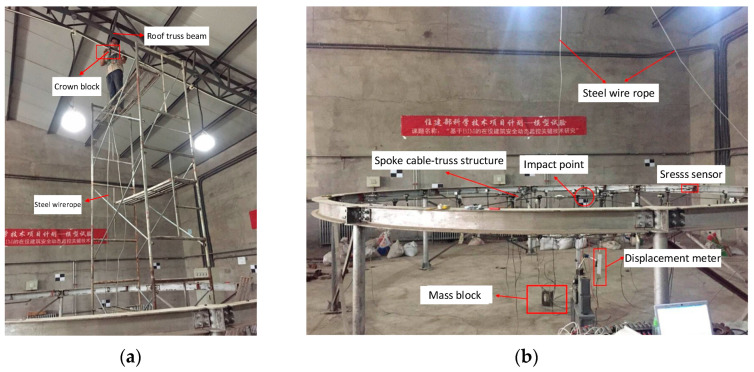
Actual model test. (**a**) Loading device for impact test; (**b**) Test structure model.

**Figure 9:**
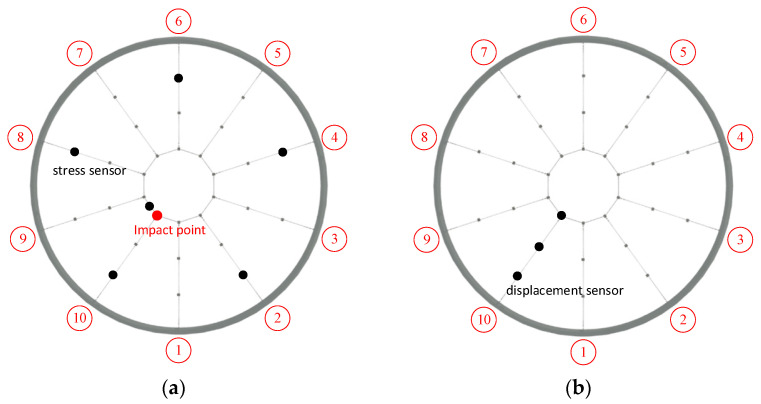
Loading device for impact test. (**a**) Cable monitoring point; (**b**) Displacement monitoring point.

This study used the experiment performed by Liu et al. [37] as a reference in this paper. Therefore, 219 working conditions were selected from the test to build a neural network model to study the impact load and the impact of different relaxation types of cable on the overall structure. This test used the drop hammer loading device, and the height of the impact test was 0.5 m, 0.75 m and 1.0 m which meets the requirements of a low-speed impact test. The drop hammer selects the same shape mass block; the mass is 5 kg and 7.5 kg. The drop hammer falls smoothly, and the drop hammer speed has strong repeatability. The drop hammer is manually driven and easy to operate. The testing device is shown in Figure 8. The drop hammer creates velocity by free fall and vertically impacts the joints of the 10th upper radial cable and the upper girdle cable of the test model. The cable force sensor and displacement meter generate voltage amplitude that changes with time, and the test selected the dynamic signal acquisition instrument to receive, display and record the signal (32 data points per microsecond). Finally, the time history curves of each cable and the node displacement are obtained. The cable force was measured by a magnetic flux sensor. A total of 12 measuring points for radial cable and girdle cable were selected (as shown in Figure 9a). The test used the displacement meter to measure the vertical displacement, and the displacement meter was set to zero scales to collect positive and negative displacement data generated by node vibration. The displacement meters are arranged at the inner, middle, and outer lower nodes of the 10th radial cable (as shown in Figure 9b).

During the test, the finite element simulation shows that relaxing the single upper and lower radial cable has little effect on the static and dynamic performance of the structure. Therefore, the cable force of a single upper and lower diameter cable relaxes by 50~100%, while the upper and lower ring cable relaxes by 20%, 30%, 40%, 50%, 60%, 70%, 80%, 90%, and 100% of the prestress applied in the finite element simulation. Some working conditions are shown in Table 2. The LM-BP algorithm in this study includes fourteen input variables and five output variables. There are 14 output neurons in the input layer. Neurons 1–5 are the relaxation residual prestress percentage of the upper radial cable. Neurons 6–10 are the relaxation residual prestress percentage of the lower radial cable. Neurons 11–12 are the relaxation residual prestress percentage of the upper and lower girdle cable. Neurons 13–14 are the height and mass of impact material, respectively. And the output layer contains five neurons. Neurons 1–2 are the peak cable forces of the upper and lower radial cables. Neurons 3–4 are the peak cable forces of the upper and lower ring cables. Neuron 5 represents the maximum displacements of the nodes under the tenth inner brace.

### 5.2. Neural Network Model

The ability of the neural network depends directly on its structure. Therefore, neural network structure optimization design is needed to establish a suitable neural network model. There are many kinds of research on the empirical formula of neural network model structures. The empirical formula method shows that the neural network model with a single hidden layer can evaluate any nonlinear relationship [35]. Table 3 shows the empirical formula for calculating the neural network model structure with the number of neurons in a single hidden layer. This paper used 14 input neurons and 5 output neurons in the prediction model, so *Ni* = 14 and *N*_0_ = 5. *Ni* and *N*_0_ are the numbers of neurons in the input layer and output layer. The structure of neural networks constructed by different formulas is very different.

The most well-recognized formula is from Zhang et al. [39]. Therefore, this paper establishes 13 LM-BP neural network models for impact response prediction, and the number of hidden layer neurons ranged from 5 to 17. *MSE* is the performance measurement standard of the neural network model. The lower the *MSE* error is, the better the model performance is. This paper considers the average *MSE* value to reduce errors, so all neural network models have been trained ten times. According to Table 4, model 9 has the best prediction performance for the impact response of prestressed steel structure, and its *MSE* (0.241) is the lowest. Therefore, this paper uses (14-13-5) as the optimal model for impact response prediction of the LM-BP neural network model.

### 5.3. Analysis of Results

This study proposes an improved BP neural network model to predict structural impact responses. Compared with the traditional model, the improved neural network algorithm has the advantages of fast training speed, high training accuracy, low parameter complexity, and good optimization effect. In addition, this study verifies the feasibility of the model based on the experimental data of prestressed steel structures. The simulation results show that the model has a high practical application value.

**Figure 10:**
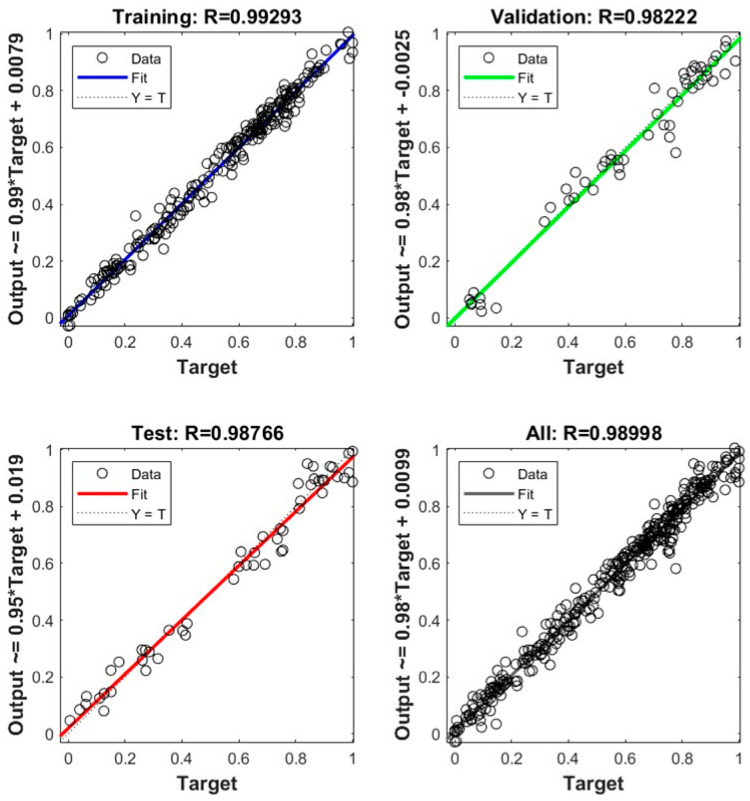
Comparative analysis of predicted data and experimental data.

**Figure 11:**
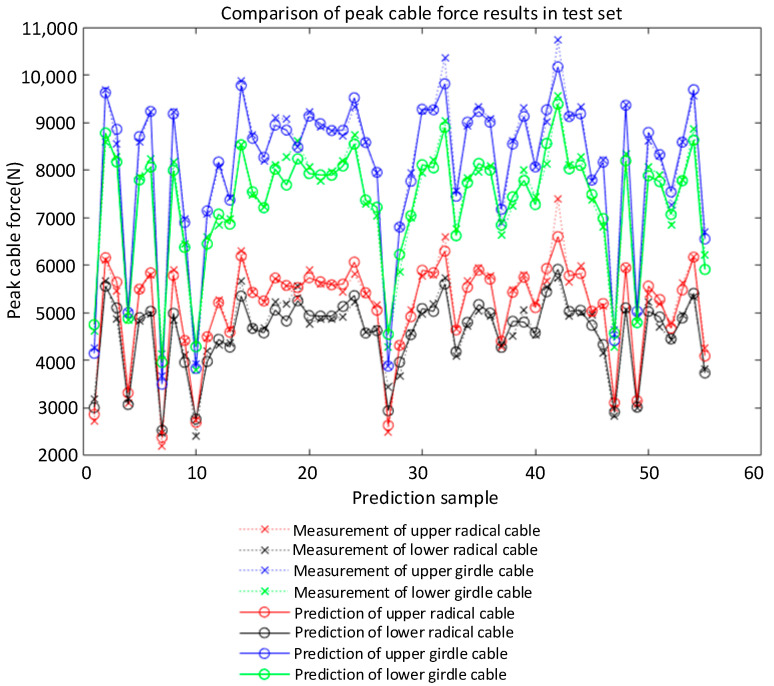
Comparison of prediction results of cable force change rate in the test set.

**Figure 12:**
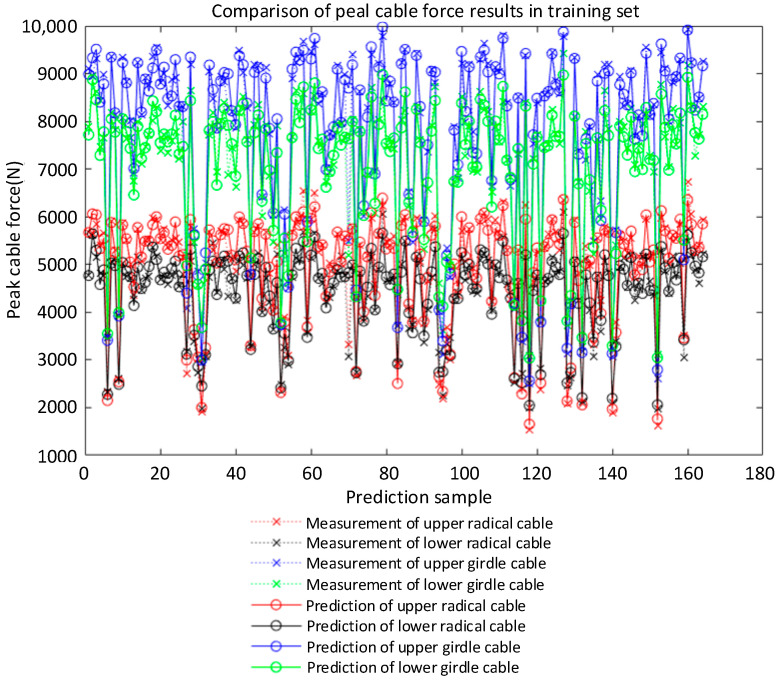
Comparison of prediction results of cable force change rate in the training set.

The R of the improved LM-BP neural network model was 0.99293 in the training stage and 0.98766 in the test stage, as shown in Figure 10. The experimental results are in good agreement with the prediction, and the relative error is within a reasonable range. The improved BP neural network model has good generalization ability and can predict the cable forces of each component in the impact test. Most of the predicted values of the neural network model are not much different from the test values, and the overall prediction effect is good (as shown from the prediction results of peak cable force in Figure 11 and Figure 12). In addition, the overall error between the actual cable force value and the predicted value fluctuates by 8%, indicating that the predicted results can ensure a higher level of reliability. At the same time, considering the complexity of the test environment and the efficiency of the neural network, this accuracy level can be regarded as an acceptable level of cable force prediction results. After analysis, the improved LM-BP neural network algorithm has high robustness in the training and testing stage, and the overall accuracy rate reaches 99.7%. The neural network algorithm is a powerful alternative method to solve a complex problem, such as calculating the cable forces under different working conditions in a case.

The test took an average of 0.7 h for each working condition (from adjusting the sleeve control cable prestress to conduct the impact test and carrying out data sorting and analysis). A total of 48 h was needed to complete the test analysis under all working conditions. The improved LM-BP neural network model can predict the results more accurately, involves less time, and has important engineering practicalities.

## 6. Discussion and Conclusions

To improve the accuracy and efficiency of the structural test and FEA, this paper has taken prestressed steel structures in the O&M stage as the research object and put forward the impact response prediction method of prestressed steel structures driven by DTs and ML. This study constructed a high-fidelity digital twin model for prestressed steel structure by using 3D laser scanning, BIM, and finite element simulation. The improved BP algorithm is used to predict the structural impact response, and the evaluation criterion is adopted to ensure the model’s accuracy.

The main contributions of this study are as follows:

(1) This study proposed a DTs-based framework for structure impact response prediction. The authors have described the details of the prediction process, including the perception layer, transmission layer, analysis layer, and service layer.
(2) This study established a digital twin model from dimensions of the physical and digital virtual entity modeling by using BIM technology, 3D scanning technology, and finite element simulation technology. This paper helps engineers save time and cost for structural modeling tasks compared with traditional methods. In addition, the proposed method also helps to avoid modeling complexity and data loss and meet the requirements of timely data exchange during OMS.
(3) This paper aims to develop an efficient analysis model which can intelligently assess the structural safety state based on the collected data. In this study, the model has been successfully applied to predict the impact response of prestressed steel structures. This study compares the prediction results of the model with the field data and verifies that the model has good accuracy and strong applicability. The results indicate that the LM-BP model can improve the efficiency and accuracy of large-scale finite element complex analysis process. This method can be successfully applied to the safety assessment of prestressed steel structures in service.

In AECO, based on the capabilities of DTs, the following directions are suggested for future research. (1) A major challenge is the stability of real-time transmission of big data in large and complex structures, and the application of 5G can be an important development direction in the future; (2) big data technologies (e.g., data mining, distributed computing, big data storage, etc.) for data analytics should be explored using the data collected from structure O&M processes; and (3) the integration of DTs with other structures’ O&M security services (e.g., cost-effective management, Structural health monitoring, structural maintenance policy-making, etc.) should be explored in further detail.

## Figures and Tables

**Table 1:** Model material selection specifications.

Member Bar	Location	Model Specification	Model Area (mm^2^)
The radial cable	Upper	6 × 7Φ8	24.6
The radial cable	Lower	6 × 19Φ10	33.3
The girdle cable	Upper	6 × 7Φ8	24.6
The girdle cable	Lower	6 × 19Φ12	49.1
Brace	Out	Φ12 × 2	62.8
Brace	Middle	Φ12 × 2	62.8
Brace	Inner	Φ12 × 2	62.8
Ring beam	Outer circle	150 × 150 × 10 × 10 I-shaped steel	4300

**Table 2:** The percentage of residual prestress of cable member relaxation.

Working Condition	Upper Radial Cable1	Upper Radial Cable2	Upper Radial Cable3	Upper Radial Cable4	Lower Radial Cable5	Lower Radial Cable1	Lower Radial Cable2	Lower Radial Cable3	Lower Radial Cable4	Lower Radial Cable5	Upper Girdle Cable	Upper Girdle Cable	Impact Height	Impact Mass
1	0.8	0.8	0.8	0.8	0.8	1	1	1	1	1	1	1	1	7.5
2	0.6	0.6	0.6	0.6	0.6	1	1	1	1	1	1	1	1	7.5
3	0.4	0.4	0.4	0.4	0.4	1	1	1	1	1	1	1	1	7.5
4	1	1	1	1	1	1	1	1	1	1	0.8	1	1	7.5
5	1	1	1	1	1	1	1	1	1	1	1	0.6	1	5
6	0.8	0.8	0.8	0.8	0.8	1	1	1	1	1	0.8	1	1	5
7	0.6	0.6	0.6	0.6	0.6	1	1	1	1	1	1	0.6	1	5
8	1	1	1	1	1	0.7	0.7	0.7	0.7	0.7	1	1	0.75	7.5
9	1	1	1	1	1	0.5	0.5	0.5	0.5	0.5	1	1	0.75	7.5
10	1	1	1	1	1	0.3	0.3	0.3	0.3	0.3	1	1	0.75	7.5
11	1	1	1	1	1	1	1	1	1	1	0.5	1	0.75	5
12	1	1	1	1	1	1	1	1	1	1	1	0.5	0.75	5
13	1	1	1	1	1	0.7	0.7	0.7	0.7	0.7	0.7	0.7	0.75	5
14	1	1	1	1	1	0.5	0.5	0.5	0.5	0.5	0.5	0.5	0.5	7.5
15	1	1	1	1	1	1	1	1	1	1	1	1	0.5	7.5
16	0.6	0.6	0.6	0.6	0.6	0.6	0.6	0.6	0.6	0.6	0.6	0.6	0.5	7.5
17	0.4	0.4	0.4	0.4	0.4	0.4	0.4	0.4	0.4	0.4	0.4	0.4	0.5	5
18	0.2	0.2	0.2	0.2	0.2	0.2	0.2	0.2	0.2	0.2	0.2	0.2	0.5	5
19	1	1	1	1	1	0.7	0.7	0.7	0.7	0.7	1	1	0.5	5

**Table 3:** Empirical formulas of the neural network structure.

Reference	Equation	Computational Results
Zhang [38]	$\sqrt{N_{i}+N_{0}}+a,a\in \left[0,10\right]$	5≤n≤15
Ripley [39]	$\left(N_{i}+N_{0}\right)/2$	n=10
Paola [40]	$\left(2+N_{0}\times N_{i}+0.5N_{0}\times \left(N_{0}^{2}+N_{i}\right)-3\right)/N_{i}+N_{0}$	n=17
Wang [41]	$2N_{i}/3$	n=10
Masters [42]	$\sqrt{N_{i}\times N_{0}}$	n=9

**Table 4:** Neural network structure and prediction results.

Model	Nodes in the Hidden Layer	Average MSE
1	5	0.768
2	6	0.751
3	7	0.659
4	8	0.595
5	9	0.515
6	10	0.449
7	11	0.333
8	12	0.286
9	13	0.241
10	14	0.372
11	15	0.414
12	16	0.537
13	17	0.659

## Data Availability

The data used to support the findings of this study are available from the corresponding author upon request.
